# Innate-like T lymphocytes in chronic liver disease

**DOI:** 10.3389/fimmu.2023.1114605

**Published:** 2023-03-15

**Authors:** Maria Papanastasatou, Mihalis Verykokakis

**Affiliations:** Biomedical Sciences Research Center Alexander Fleming, Institute for Fundamental Biomedical Research, Vari, Greece

**Keywords:** innate-like T cells, NAFLD, NASH, HCC, chronic inflammation

## Abstract

In addition to its metabolic activities, it is now clear that the liver hosts a number of diverse immune cell types that control tissue homeostasis. Foremost among these are innate-like T lymphocytes, including natural killer T (NKT) and mucosal-associated innate T (MAIT) cells, which are a population of specialized T cells with innate characteristics that express semi-invariant T cell receptors with non-peptide antigen specificity. As primary liver residents, innate-like T cells have been associated with immune tolerance in the liver, but also with a number of hepatic diseases. Here, we focus on the biology of NKT and MAIT cells and how they operate during the course of chronic inflammatory diseases that eventually lead to hepatocellular carcinoma.

## 1 Introduction

Traditionally, the liver is considered as the central metabolic organ of the body that performs numerous functions, including carbohydrate metabolism, nutrient uptake and storage, biosynthesis of various biochemical compounds, lipid metabolism, and detoxification (1). In healthy individuals, the liver is constantly exposed to several gut-derived foreign microbial and dietary antigens, which may be recognized by receptors expressed on hepatic cells, thus initiating a pro-inflammatory response. However, this type of response during homeostasis, if uncontrolled, would be devastating for the organism, because it would eventually lead to liver injury and related pathologies (2). The liver has developed a distinct immunological environment that enables constant screening of foreign products without generating an excessive immune response. These tolerogenic properties of the liver are tightly regulated by a complex network of liver-resident immune cells. Failure to resolve inflammation is linked with the development of liver damage, characterized initially by steatosis and hepatic fibrosis, which may progress to cirrhosis and eventually to hepatocellular carcinoma (HCC) (3). Therefore, in addition to its major metabolic functions, the adult liver exhibits essential immunological features, including induction of immune tolerance, immuno-surveillance and innate and adaptive immune cell residency.

The adult liver is populated by several cells with innate and adaptive immune properties (4). Innate immune cells are of myeloid lineage that develop in the bone marrow, before migrating to the liver. These cells include Kuppfer cells, granulocytes, and neutrophils, which express germline-encoded receptors and respond quickly after microbial infection, thus controlling the early phase of an immune response. Adaptive immune cells are of lymphoid lineage and include B and T cells, which derive from the bone marrow and the thymus, respectively. These cells express a diverse repertoire of rearranged receptors that recognize foreign antigens with great specificity, although, due to clonal expansion, the adaptive immune response peaks several days after pathogenic insult. Despite the developmental and functional distinctions between innate and adaptive immune cells, there are cells that cross these traditional boundaries (5). Innate lymphoid cells (ILCs), including natural killer (NK) cells, develop from common lymphoid progenitors, however they exist in a primed state and recognize pathogens through germline encoded receptors. In addition, innate-like T cells, including NKT, Mucosal-Associated Innate T (MAIT) and γδ T cells, are characterized by constitutive expression of NK and activated T cell markers, and are programmed to produce large amounts of cytokines quickly after antigenic encounter. In this review, we focus on how innate-like T cells function in inflammatory liver diseases.

## 2 Innate properties of innate-like T cells

Our knowledge on the biology of innate-like T cells derives mainly from Type I or invariant NKT (iNKT) cells, which have been studied extensively in the past 30 years (6). Similar to conventional T cells, iNKT cells develop in the thymus from common T lymphocyte progenitors in a process that is dependent on antigen receptor rearrangements and production of a functional T cell receptor (TCR) (7, 8). However, their TCR is rather oligoclonal and is activated by a range of lipid antigens, presented by the non-classical major histocompatibility complex I (MHCI)-like molecule CD1D, which is expressed in antigen-presenting cells, such as Kuppfer cells, dendritic cells, and hepatocytes (9). In addition to TCR expression, iNKT cells constitutively express several cytokine receptors and Toll-like receptors at the steady state; as a consequence, they have the potential to respond to danger signals and/or cytokines produced by other cell types during liver damage, even in the absence of TCR-CD1D-antigen interactions (10). iNKT cells respond within hours after stimulation with the production of a broad array of cytokines, including interleukin (IL)-4, IL-17, IL-10, and interferon gamma (IFNγ) (11), which in turn may stimulate T and B cells or recruit neutrophils, monocytes, and myeloid cells in the site of damage. Therefore, iNKT cells orchestrate the immune response by bridging the functions of innate and adaptive immune cells.

In addition, another population of CD1D-dependent NKT cells with more diverse TCR usage, including non-canonical Vα3.2Jα9/Vb8 and Vα8/Vβ8 rearrangements has been described, albeit in much less detail (12). These Type II or diverse NKTs (dNKT) preferentially recognize sulfatide; sulfatide versus glycolipid recognition is a critical distinguishing feature between these NKT subsets, and CD1D tetramers loaded with the protypical iNKT ligand αGalactosylCeramide (αGalCer) or αGalCer analogs (i.e PBS57) are used to specifically detect iNKT cells (13, 14). Despite their similarities, Type I and Type II NKT cells have distinct functions and, in some cases, they suppress each other during an immune response (15). Unfortunately, due to the lack of reagents that specifically detect dNKT cells, studies regarding the functions of this cell type have been limited.

The hybrid nature of iNKT cells is also reflected in their core transcriptional program, which resembles both that of the adaptive and innate immune cells (16). Interestingly, this innate-like program is established during their distinct thymic development, prior to foreign antigen encounter. iNKT cells arise from CD4^+^CD8^+^ (double positive, DP) precursors expressing the characteristic iNKT TCRα (Vα14-Jα18 in mice, Vα24-Jα18 in humans), after random genomic rearrangement of the *Tcra* locus, which pairs with a limited number of Vβ chains (7). Positive iNKT TCR selection is mediated by homotypic DP-DP interactions, through TCR and SLAM receptor signaling (17). This unique positive selection pathway leads to the induction of PLZF expression, a transcription factor of the BTB-POZ family, which is both required and sufficient to establish their innate properties to iNKT cells (18, 19).

Concomitant with their development, committed iNKT cell precursors undergo massive proliferative expansion and differentiate intrathymically in three distinct subsets, defined according to the expression of the T helper (Th) signature transcription factors TBET, GATA3, and RORγt (20). Consistent with their Th1-associated properties, NKT1 cells express and are dependent on TBET; Th2-like NKT2 cells express high levels of GATA3 while Th17-like NKT17 cells are RORγt^+^. Notably, the polarization of the iNKT effector subsets is not as absolute as that of the conventional CD4 T cells, since iNKT cells have the unique ability to produce both Th1 and Th2-associated cytokines at the single cell level.

Similar to iNKT cells, MAIT cells are characterized by a poised effector state, which is pre-programmed during their step-wise thymic development (21). MAIT cells express a semi-invariant TCR, consisting of a canonical Vα19-Jα33 chain in mice paired with a limited number of Vβ chains, which is positively selected through DP-DP interactions (22, 23). MAIT cells rely on the expression of PLZF for their thymic maturation and acquisition of their innate-like properties (24). In addition, they are enriched in mucosal sites, particularly in the gut and the liver, although they are also found in tissues where conventional T cell are present. However, MAIT cells have significant differences compared to iNKT cells. Foremost, they recognize microbial vitamin B metabolites presented in the context of the highly conserved non-polymorphic MHCI-like molecule MR1 (25, 26). While they exist in very low numbers in mice, MAIT cells outnumber iNKT cells in humans, where they may consist up to 50% of T cells in the liver and 10% in the blood. Functionally, the majority of MAIT cells expresses RORγt and IL-17 (MAIT17), whereas only few MAIT cells are TBET^+^ expressing IFNγ (MAIT1); MAIT2 cells are very rare, if any in mice (21, 24). Recently, specific MR1-tetramers loaded with 5-OP-RU have been developed and revolutionized the study of MAIT cells (27).

## 3 Innate-like T cells in liver diseases

Both iNKT and MAIT cells are enriched in the murine and human liver compared to other anatomical locations. Given that both these cell types sense dietary antigens and/or changes in endogenous metabolic products, through their TCR, it is not surprising that they regulate, either in a protective or harmful manner, multiple functions in the liver, including, hepatic injury, inflammation, fibrogenesis and tumorigenesis (28) associated with chronic inflammation related to viral infection or metabolic disorders. In this review, we attempt to delineate the complex functions of innate-like T cells in liver inflammation, fibrosis and cancer.

### 3.1 Hepatitis B virus and hepatitis C virus

Hepatitis B and hepatitis C viruses are the most common causes for chronic viral hepatitis. HBV is a member of the Hepadnaviridae family with a small DNA molecule and features similar to retroviruses (29). While more than 2 million people are infected with HBV wordwide (30), HBV infection may go undetected or with mild illness, which resolves within a few weeks, for most patients. However, in other people HBV may cause lifelong complications and approximately 15-40% of untreated chronic HBV patients progress to liver cirrhosis and may develop liver cancer (31). Importantly, development of prophylactic vaccines for all ages against HBV have helped to build immunity and reduce the incidence of hepatitis B globally. Diagnosis of chronic HBV is based on histological and biochemical features, measurement of HBs Ag and expression level of HBV DNA. The treatment of chronic HBV infection must limit HBV replication and liver inflammation, and prevent development of cirrhosis and liver cancer. The most commonly used therapy against chronic HBV is treatment with pegylated IFNα (32). HCV is an RNA virus (33) that belongs to the Flaviviridae family and infects more than 71 million people in the West with high mortality due to the ensuing cirrhosis and liver cancer (34). Detection of HCV antibodies and measurement of HCV RNA are common methods to diagnose HCV infection (35). The most effective therapy for chronic HCV patients is application of IFN-free regiments according to stage therapy (35), while direct-acting antivirals are used in late-stage chronic HCV patients (36). Both HBV and HCV trigger strong innate and adaptive immune responses accompanied by robust IFN response, mediated in part by activation of liver resident NKT and MAIT cells. However, activation of, at least, iNKT cells has been linked to liver damage, while it is currently unclear whether iNKT and MAIT cells directly recognize HBV and HCV ligands, either through their TCRs or Toll-like receptors. Therefore, how innate-like T cells collectively contribute to viral clearance in the liver is still under intense investigation.

#### 3.1.1 MAIT cells in chronic HBV and HCV

**Figure 1:**
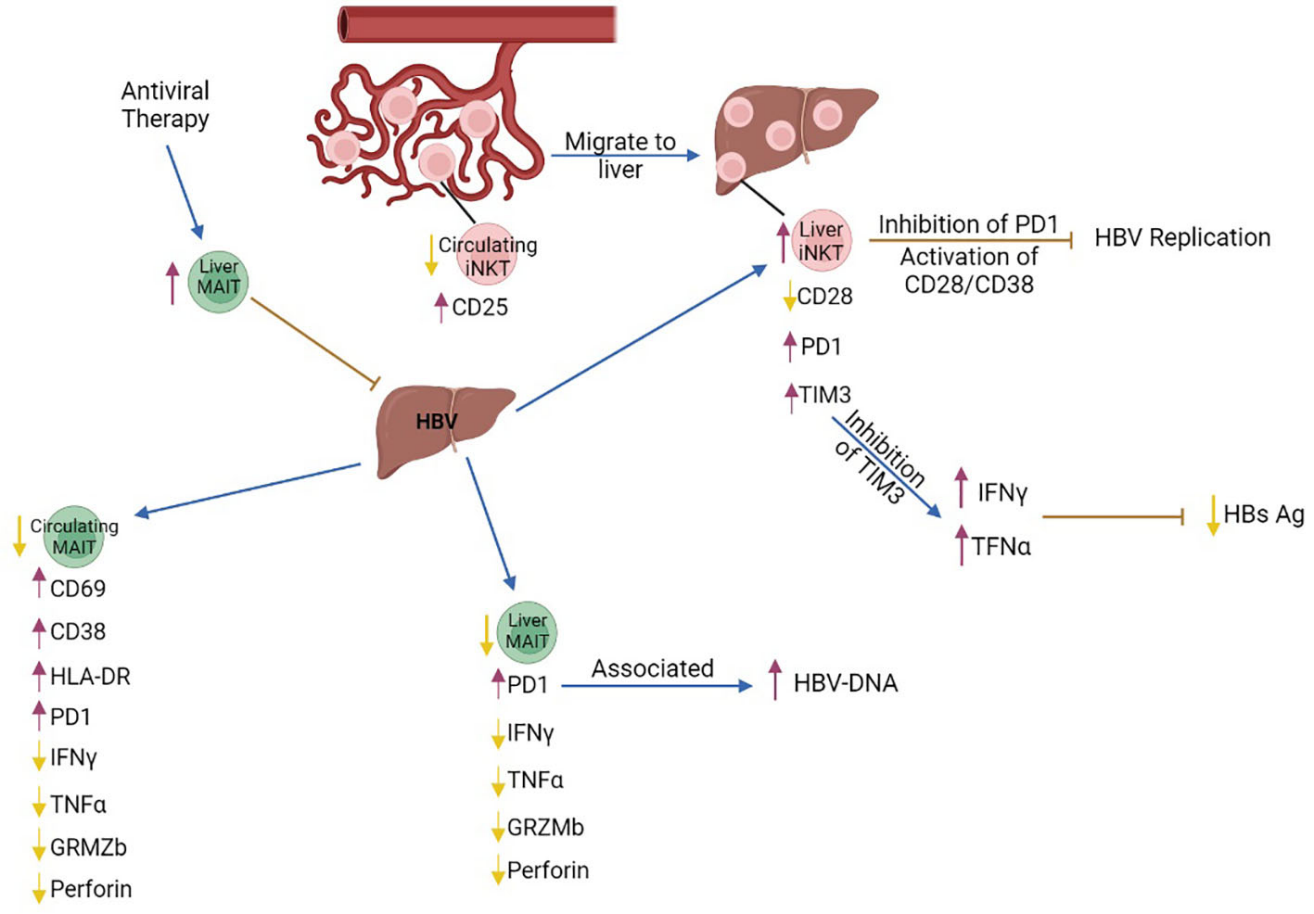
The role of MAIT and iNKT cells in chronic HBV infection. Intrahepatic iNKT cells highly express the exhaustion markers PD1 and TIM3 while expression of the activated marker CD28 is decreased. Inhibition of PD1 and activation of the CD28/CD38 signaling pathway in liver iNKT cells leads to inhibition of HBV replication. Inhibition of TIM3 expression in liver iNKT cells leads to increased production of IFNγ, which increases production of type I interferons, IFNα and IFNβ, leading to reduction of HBs Ag levels. Although the number of circulating MAIT cells is decreased, the remaining cells express the activation markers CD69 and HLA-DR, and the chronic activation marker CD38, while production of IFNγ, TNFα and Granzyme B is decreased. The number of liver MAIT cells is lower in chronic HBV, and they are characterized by increased expression of PD1. PD1^+^MAIT cells are associated with an increase in HBV-DNA levels. Antiviral therapy increased the frequency of MAIT cells in the liver and contributed to viral clearance. purple arrows, increase; yellow arrows, decrease; brown arrows, inhibition; blue arrows, promotion.

The frequency and number of MAIT cells is significantly decreased in the peripheral blood and the liver of patients with chronic hepatitis B (37–42) and is associated with middle/late stages of the disease compared to early stages. Notably, the decrease in circulating MAIT cells indicates poor prognosis for patients with chronic HBV (40). Peripheral MAIT cells express higher levels of the activation markers CD69 (37), CD38 (37–39, 42), HLA-DR (37, 38) than intrahepatic MAIT cells (37) indicating that circulating MAIT cells are more activated than MAIT cells in the liver. In addition, blood and liver MAIT cells from patients express high levels of PD1, compared to healthy individuals (39, 42), which is directly associated with increased plasma levels of HBV-DNA (39), indicating that PD1^+^MAIT cells are dysfunctional in patients with chronic HBV infection. Consistent with that, MAIT cells from chronically HBV infected patients produce lower amounts of IFNγ, TNFα, granzyme B, and perforin after *ex vivo* stimulation, compared to healthy controls (37–39, 41). Importantly, the frequency and the number of MAIT cells is restored after anti-viral therapy in patients that survived compared to those that succumbed to disease (40) (**Figure 1**). Consistent with these findings, MAIT cells exhibit strong and direct cytotoxicity against HBV-infected hepatocytes (41), rendering them potential targets in anti-viral therapeutic strategies.

**Figure 2:**
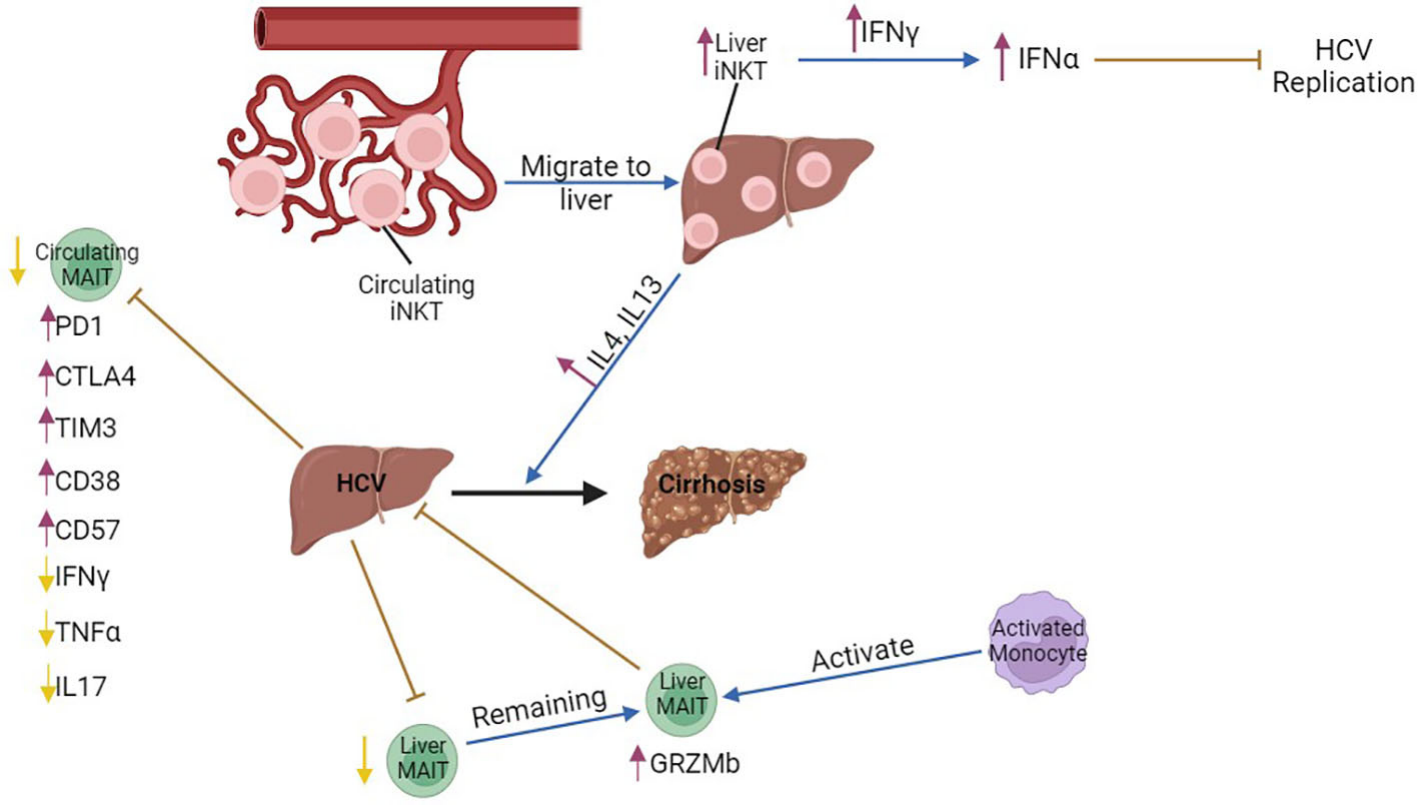
The role of MAIT and iNKT cells in chronic HCV infection. The number of circulating iNKT cells is lower compared to that of hepatic iNKTs in chronic HBV, possibly indicating that peripheral iNKT cells migrate from the circulation to the liver and produce increased amounts of IFNγ during transition to cirrhosis. Liver iNKT cells show increased production of type I interferons, IFNα and IFNβ, through increased production of IFNγ and, thus, inhibit HCV replication in the acute phase of the disease. Circulating MAIT cells are decreased, and express the exhaustion markers PD1, CTLA4 and the chronic activation marker CD38. However, they produce lower amounts of IFNγ, TNFα, and IL17. Liver MAIT cells are decreased, while the remaining MAIT cells are activated and inhibit chronic HCV infection. Hepatic MAIT cells are activated by monocytes. purple arrows, increase; yellow arrows, decrease; brown arrows, inhibition; blue arrows, promotion, Black arrow, progression of the disease.

Similar to chronic HBV infection, the frequency and the number of circulating and intrahepatic MAIT cells is reduced in patients with chronic HCV infection (43–49), although MAIT cells are enriched in the liver compared to the blood of chronic HCV patients (46). This reduction in MAIT cell number is independent of the disease stage (44). Peripheral MAIT cells are characterized by an exhausted phenotype, as shown by the increased expression of exhaustion markers PD1, CTLA4 and TIM3 (44, 45) and the chronic activation marker CD38 (45, 48). In addition, MAIT cells produced reduced levels of IFNγ, TNFα, and IL17 after E. coli stimulation, although production of these cytokines was normal after IL12/IL18 stimulation (44, 46), indicating impaired TCR-dependent MAIT cell responses. The frequency of circulating CD57^+^ (senescence marker) MAIT cells is increased, providing additional evidence that MAIT cells are dysfunctional in chronic HCV patients (45). Activation/exhaustion of intrahepatic MAIT cells is mediated through liver monocyte-derived cytokines in patients with chronic HCV infection (46). In addition, MAIT cells are more cytotoxic (46) due to the increased production of granzyme B (44). IFN-free therapy against chronic HCV infection leads to clearance of the virus, although the frequency of MAIT cells does not recover (44), but the number of CD8^+^ T and NK cells is increased (50). IFNα-based therapies increase the frequency and activation of MAIT cells in the liver (43) leading to partial resolution of liver inflammation (46). Taken together, these data suggest that the frequency and number of MAIT cells is inversely correlated with liver inflammation and fibrosis in chronic HCV infection (**Figure 2**), while the function of the remaining MAIT cells is impaired. It is, thus, possible that this exhausted phenotype contributes to the chronicity of viral infection, although studies that examine the function of MAIT cells in acute viral hepatitis are currently lacking.

#### 3.1.2 iNKT cells in chronic HBV and HCV

iNKT cell number is significantly increased in the liver of HBV transgenic mice with acute hepatitis. HBV-infected hepatocytes presented lipid antigens to liver iNKT cells, through CD1D presentation, thus leading to their activation and immediate production of anti-viral IFNγ (51). IFNγ inhibited proliferation of infected hepatocytes and enhanced innate and adaptive immune responses, including the activity of cytolytic cells (51, 52). Importantly, αGalCer-activated iNKT cells directly inhibited HBV replication in the liver of HBV-transgenic mice (53), eventually limiting HBV infection through various mechanisms. Although iNKT cells contribute to the control of acute HBV infection, their functionality and number are compromised in patients and HBV transgenic mice with chronic HBV infection, as indicated by PD1 upregulation and CD28 downregulation, and their impaired ability to produce IFNγ (54, 55). Inhibition of PD1 and activation of the CD28/CD80 pathway in iNKT cells inhibited HBV replication (54). iNKT cells expressed high levels of TIM3 in HBV transgenic mice and inhibition of TIM3 restored production of IFNγ and TNFα, resulting in a reduction of serum HBs Ag and inhibition of HBV replication (56) (**Figure 1**).

Although the number of blood iNKT cells is reduced in patients with chronic HBV-related cirrhosis, circulating iNKT cells are hyperactive during transition from chronic HBV to cirrhosis, as shown by increased expression of CD25 and several cytokines (57). This reduction in blood iNKT cell number was probably due to iNKT migration to the liver, because proliferation or apoptosis remained unaffected. Isolated peripheral iNKT cells from these patients were able to activate an HSC cell line *in vitro* and promoted proliferation of hepatocytes, indicating that in a chronic HBV inflammatory background, iNKT cells may eventually contribute to progression to liver cirrhosis (57, 58) (**Figure 1**).

The frequency of iNKT cells was significantly reduced in the blood of HCV-seropositive patients (59, 60), while their frequency increased in the liver (61), possibly indicating that iNKT cells migrate from the periphery to the liver. Hepatic iNKT cells from patients with chronic HCV infection produced high levels of IFNγ, while they did not produce Th2-related cytokines, such as IL4 or IL13. Interestingly, iNKT cells from patients with HCV-related cirrhosis showed a marked increase in the production of pro-fibrotic IL4 and IL13, suggesting that iNKT cell effector functions are modified during progression of chronic viral hepatitis to cirrhosis. In addition, expression of CD1D is upregulated in cirrhotic livers, which indicates that continuous lipid presentation may induce a switch in cytokine production from iNKT cells during the course of chronic viral hepatitis that contributes to the development of hepatic cirrhosis (61). Nonetheless, in a humanized mouse model of HCV infection, IFNα treatment triggered production of IFNγ by iNKT cells, which lead to inhibition of HCV replication (62) (**Figure 2**).

### 3.2 NAFLD-NASH

Non-alcoholic fatty liver disease (NAFLD) is a heterogeneous condition characterized by a build-up of extra fat in the liver, which is not caused by alcohol consumption (63, 64). Histologically, NAFLD is classified in two types: non-alcoholic fatty liver (NAFL), characterized by simple hepatic steatosis and it is not accompanied by hepatic inflammation, and non-alcoholic steatohepatitis (NASH), which is more severe than NAFL and is accompanied by hepatocyte damage and chronic inflammation that may eventually lead to cirrhosis and hepatocellular carcinoma (65, 66). Although the background of steatosis increases the risk of NASH development, NAFL does not necessarily transition to NASH (67). People that develop NASH are usually overweight or have been diagnosed with diabetes or obesity. While the exact etiology of NASH is unclear, it is currently acknowledged that multiple triggers, including, *de novo* lipogenesis, hepatocyte death and the ensuing liver injury, and inflammation may contribute to NASH onset, fibrosis, and NASH/HCC transition (68). However, NASH may develop due to other causes, such as high cholesterol, fat accumulation, and metabolic syndrome (69). Importantly, patients with NASH may be asymptomatic for years before progressing to fibrosis and cirrhosis, which challenges timely diagnosis and treatment (70). Unfortunately, although limiting NAFLD and identifying patients at risk to develop NASH/HCC are major health challenges, currently there are no approved therapies against NASH (70), whereas reliable predictive biomarkers of disease progression are also lacking. The undoubtful role of inflammation in the pathogenetic processes of NAFLD indicates that liver-resident immune cells that recognize dietary metabolites, such as MAIT and iNKT cells, may modulate development and progression of NAFLD (4).

Several mouse models and diets have been developed to experimentally study NAFLD and NASH. The most commonly used diet is methionine/choline-deficient diet (MCD diet) that contains high sucrose and fat but lacks methionine and choline, which are necessary for mitochondrial oxidation and low-density lipoprotein synthesis. MCD leads to oxidative stress, liposynthesis and eventually, to hepatic steatosis (71). Choline-deficient diet (CDD), which is another dietary scheme used to study liver disease, induces fat accumulation in the liver (72) without affecting the adipose tissue (73) and it does not cause severe steatohepatitis (73). In addition, another diet that is used to study NAFLD is high-fat diet (HF). Mice fed with HF become obese and develop hyperinsulinemia, hyperglycemia, hypertension, and liver damage, a phenotype that is similar with the phenotype of NAFLD patients (74, 75). Combination diets, such as CDD with HF (CD-HFD) are also popular, because the added fat helps to maintain euglycemia and mitigate weight loss in mice (76). Finally, the high-trans fatty acid and high-carbohydrate diet (HFHC) closely recapitulates human NASH development following a similar process to liver fibrosis (77).

Liver fibrosis may also be induced with the use of chemical compounds, which act as hepatotoxic factors. Administration of carbon tetrachloride (CCL_4_) through various routes (intraperitoneal, inhalation, etc) is commonly used to promote hepatotoxicity. Cytochrome P450 superfamily members metabolize CCL_4_ into the active ingredient trichloromethyl radical (CCL_3_^*^) that impairs multiple cellular processes due to its reaction with proteins, nucleic acids, and lipids leading to fatty regeneration and steatosis (78). CCL_4_-induced NAFLD is initially characterized by hepatic damage including inflammation and fibrosis, which may develop to cirrhosis and finally, HCC (adducts in DNA and causes mutations) (78).

#### 3.2.1 MAIT cells in NAFLD and NASH

MAIT cells recognize antigens presented by a molecule called MR1, which is expressed in antigen presenting cells (APCs) (79). The frequency of MAIT cells was increased in patients’ NAFLD livers (80), whereas it was decreased in patients’ blood (80–82); interestingly, patients with cirrhosis showed decreased MAIT cell frequency in the liver (81), indicating that MAIT cell accumulation in the liver may depend on the stage of the disease. Notably, in cirrhotic livers, MAIT cells were relocated from the sinusoids to the fibrotic septa, possibly indicating interactions of MAIT cells with fibrogenic cells. Indeed, activated MAIT cells increased proliferation of hepatic myofibroblasts, which accumulate in sites of liver injury, in an MR1-dependent way, while they also stimulated secretion of pro-inflammatory cytokines by hepatic fibrogenic cells and macrophages through TNFα (81). MAIT cells from blood samples showed enhanced activation, according to the expression of the activation markers CD69 and CD25, and increased expression of the chemokine receptor CXCR6 (80, 81). CXCR6 is involved in the recruitment of immune cells in the liver (83) and the increased levels of CXCR6 in MAIT cells suggest that circulating MAIT cells may migrate to the liver in patients with NAFLD.

**Figure 3:**
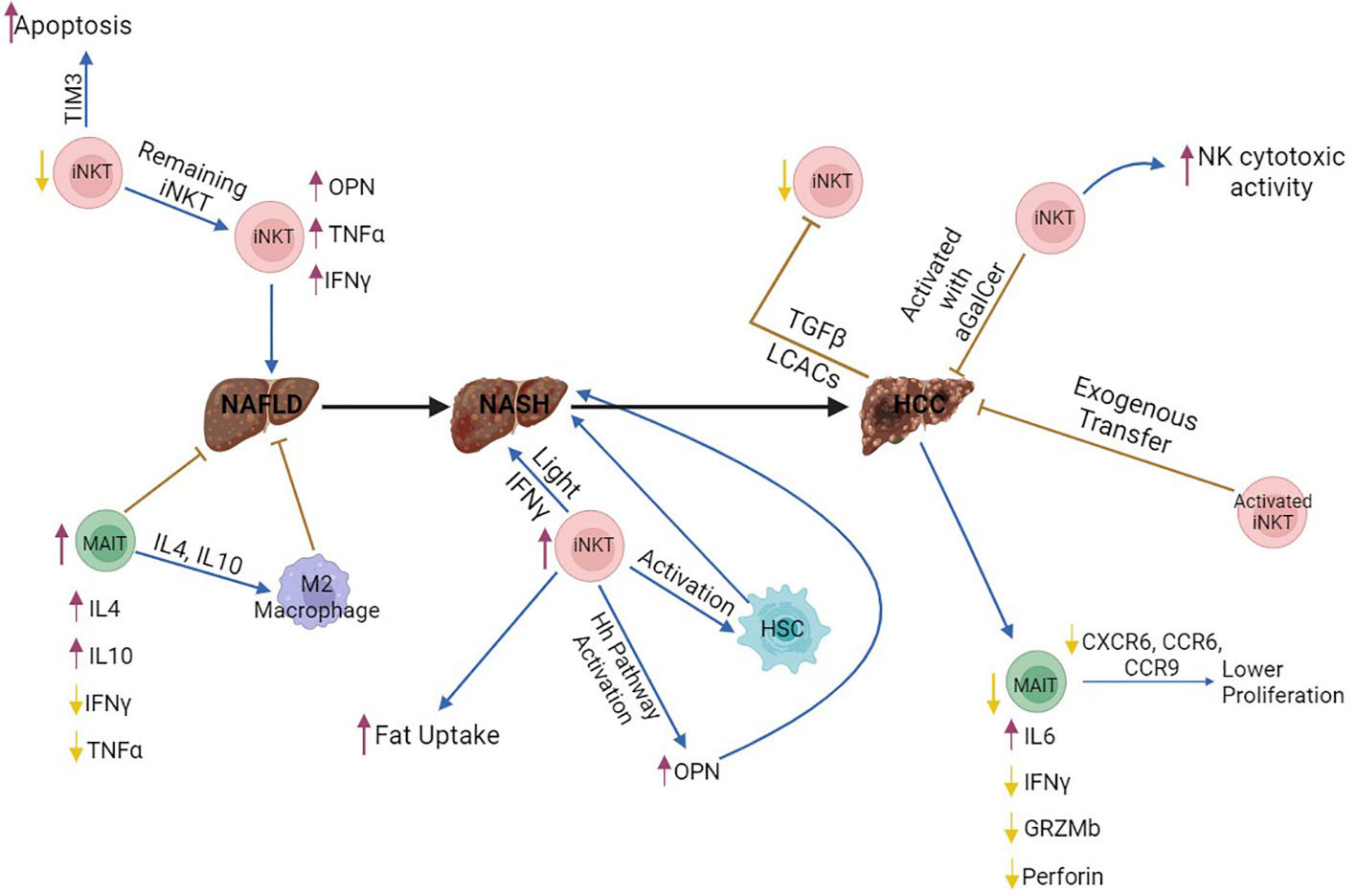
The role of MAIT and iNKT cells in NAFLD, NASH and HCC. Invariant NKT (iNKT) cells produce increased amounts of fibrogenic factors, such as OPN, and pro-inflammatory cytokines, including IFNγ and TNFα, thus enhancing liver damage and NAFLD onset. In addition, iNKT cells promote NASH development through enhanced fat uptake by hepatocytes, activation of hepatic stellate cells, and production of Light, which enhances activation of CD8^+^ T cells. In contrast, activation of iNKT cells with aGalCer or exogenous transfer of iNKT cells suppresses HCC development and enhances the function of NK cells. However, HCC cells may suppress this anti-oncogenic function of iNKT cells through production of TGFβ and long-chain acylcarnitines, which are directly recognized by iNKT cells, thus leading to impaired secretion of IFNγ and TNFα. Therefore, iNKT cells may promote initiation of liver tumorigenesis through NAFLD and NASH development, while they may be able to fight established tumors. MAIT cells contribute to the resolution of NAFLD through IL4 and IL10 production, which drive M2 polarization in macrophages. However, MAIT cell number is lower in HCC, and these cells are dysfunctional, because they produce lower amounts of Granzyme B, Perforin, and IFNγ. It is still unclear how iNKT and MAIT cells interact with each other in the liver, during homeostasis or disease. All figures were created in BioRender.com purple arrows, increase; yellow arrows, decrease; brown arrows, inhibition; blue arrows, promotion; Black arrow, promotion of the disease.

MAIT cells from the liver of mice with MCD-induced NAFLD stimulated with Phorbol 12-myristate 13-acetate (PMA) and ionomycin produced increased levels of anti-inflammatory cytokines, such as IL4 and IL10, and lower levels of the pro-inflammatory cytokines IFNγ and TNFα (80, 84, 85), indicating a switch to Th2 cytokine production. *Mr1* ^−/−^ mice that lack MAIT cells, developed NAFLD with more severe hepatic steatosis and increased lipid accumulation (80), accompanied with increased gene expression of pro-inflammatory cytokines, such as TNFα, and increased number of M1 macrophages (80). However, *Mr1^−/−^* mice were protected from CCL_4_-induced liver fibrosis, although liver damage was comparable to their *WT* counteparts (81). M1 macrophage polarization was enhanced by pro-inflammatory cytokines (IFNγ, TNFα), while anti-inflammatory cytokines (IL4, IL10) lead to differentiation of M2 macrophages (86). M2 macrophages are associated with reduced liver damage in mice and humans with NAFLD (66) and M2 differentiation is reduced in *Mr1^−/−^* mice (80, 84), while MAIT cells isolated from human NAFLD patients promoted M2 polarization, probably through IL4 production (80, 84). Taken together, these results indicate that MAIT cells regulate the fate of macrophages and contribute to the resolution of early inflammation during NAFLD development, although they may promote liver fibrosis when disease progresses to cirrhosis (**Figure 3**).

#### 3.2.2 iNKT cells in NAFLD and NASH

Similar to MAIT cells, an accumulation of iNKT cells in the liver (87) and reduction of iNKT cells in patients’ blood (82, 88) has been observed in patients with NAFLD. The frequency of hepatic iNKT cells is positively correlated with steatosis severity (89). Hepatic iNKT cells from NAFLD patients are in an activated status, according to the increased expression of CD69 (87). Isolation of human hepatic iNKT cells from patients with NAFLD and stimulation with α-GalCer (89) *in vitro* showed increased production of IFNγ (87, 89) compared to healthy individuals. Increased hepatic expression of CD1d has been observed in NAFLD patients (87, 88), indicating that presentation of lipid antigens to iNKT cells may be enhanced, leading to increased activation of iNKT cells.

Murine models of obesity and NAFLD using the leptin-deficient mice *ob/ob*, showed that development and progression of NAFLD was associated with a reduction in iNKT cell number (90). Adoptive transfer of iNKT cells in *ob/ob* mice led to decreased hepatic steatosis and improved glucose tolerance (91). Administration of choline-deficient diet (CDD) in mice, which contributes to NAFLD development, promoted steatosis characterized by loss of iNKT cells (84). In addition, in a high-fat (HF) diet murine model, mice developed NAFLD and the frequency of iNKT cells decreased specifically in the liver (85, 92), probably due to increased apoptosis; however, the remaining iNKT cells showed increased production of IFNγ and TNFα (92). In the HF diet model, the increased apoptosis of iNKT cells contributed to insulin resistance and hepatic steatosis (92). A possible mechanism that leads to iNKT cell apoptosis is through the Tim-3/Galectin-9 signaling pathway. Tim-3 expression was significantly increased in iNKT cells, and the upregulation of Tim-3 was correlated with progression of steatosis (93). Interestingly, Tim-3^+^ iNKT cells were more prone to apoptosis compared to Tim-3^−^ iNKT cells.

iNKT cell number was increased in the liver of mice with NASH. iNKT cells expanded in mice fed with MCD diet (94), characterized by an increased production of IFNγ, OPN, and IL15 (94, 95). Although the early phase of steatohepatitis is associated with lower frequency of iNKT cells (84, 92), iNKT cell expansion with concomitant up-regulation of IL15 indicates advanced NASH (84, 94). In addition, in MCD-fed mice, progression of NASH was associated with activation of the Hh pathway (96), which lead to the production of Hh-regulated fibrogenic factors (OPN) and the chemokine CXCL16 (iNKT cell chemoattractant) (97), resulting in the recruitment and accumulation of iNKT cells in the liver (87, 88). Administration of MCD diet in *CD1d^−/−^* mice that lack iNKT cells, showed significant attenuation of fibrogenesis and iNKT cell depletion rescued from fibrosis and NASH (88). In CD-HFD-fed mice, the increased number of iNKT cells was associated with enhanced uptake of fat by hepatocytes, activation of hepatic stellate cells (HSC) and induced steatosis (98). Increased numbers and activation of T cells (CD4^+^, CD8^+^) was observed in CD-HFD mice with enhanced secretion of IL17 by CD4^+^ T cells and TNFα by CD8^+^ T cells (98). *β2m^−/−^* mice, which lack CD8^+^ and NKT cells, fed with CD-HFD showed no liver damage, fibrosis and NASH and lower levels of cholesterol and triglyceride compared to CD-HFD-fed *WT* mice. Depletion of CD8^+^ T cells using anti-CD8 rescued from CD-HFD-induced liver damage with no change in cholesterol levels (98), but iNKT cells were not affected and promoted fat uptake from hepatocytes. light signaling in hepatocytes promotes lipid uptake and *Light^−/−^* CD-HFD-fed mice showed reduced liver damage. Interestingly, iNKT cells were decreased in *Light^−/−^* CD-HFD-fed mice, while the number of CD8^+^ T cells was not affected (98) compared with CD-HFD-fed *WT* mice. Blocking LTβR signaling in CD-HFD-fed *WT* mice did not rescue from liver damage. The phenotype of CD-HFD-fed *Light^−/−^* mice was associated with reduction of iNKT cell number. Therefore, iNKT cells contribute to initiation of liver damage, interact with CD8^+^ T cells through light and encumber liver damage in CD-HFD mice. *CD1d^−/−^* mice fed with HFHC were protected from NASH, did not gain weight, had normal levels of ALT (liver damage marker), fasting glucose, a-SMA (fibrosis marker) and lower or no steatosis (99). Importantly, iNKT cells were enriched in patients with NASH (88, 98,). Therefore, all this evidence supports that iNKT cells promote initiation and progression of liver fibrosis (**Figure 3**).

### 3.3 Hepatocellular carcinoma

Liver cancer is a main leading cause of death in the West (100), mainly due to the fact that there are no biomarkers that allow for early diagnosis of this disease (101). HCC is the most common type of liver cancer, which accounts for ~80% of cases (102, 103). A variety of immune cells are associated with HCC such as iNKT cells, cytotoxic CD8 T cells, helper CD4 T cells, regulatory T cells (Treg), myeloid-derived suppressor cells (MDSC), natural killer cells (NK), and dendritic cells (DCs) (104). Interestingly, the ability of DCs to become activated and present antigens to T cells is reduced in HCC, due to impaired cytokine production and reduced HLA expression, thus leading to weak T cell responses (105, 106). In addition, MDSCs play an important role in the development of HCC, as they suppress functions of immune cells (107) and promote expansion of Tregs (108, 109). In HCC, Tregs suppress secretion of IFNγ by T cells, thus negatively affecting T cell proliferation (110) as well as responses from NK cells (111). Additionally, CD8 T cells have normal cytotoxic functions, but they are dysfunctional to a large extent, while at the same time the number of CD4 T cells is significantly reduced (111). However, the role of MAIT and iNKT cells in HCC is not well understood.

#### 3.3.1 MAIT cells in HCC

Studies using human blood and HCC samples showed that MAIT cells were significantly decreased in HCC compared with adjacent tissue (112, 113). Circulating MAIT cells from the blood expressed higher levels of the exhaustion marker PD-1, suggesting a deficiency of MAIT cells in HCC patients (114). Normally, MAIT cells produce Th1- and Th17-related cytokines (112, 115, 116). Stimulation of MAIT cells isolated from HCC samples and blood with IL-2/IL-18 and PMA/ionomycin for 24 hours, showed an increased production of IL-6 and decreased IFNγ, indicating that the function of MAIT cells is impaired (114). In addition, stimulation of HCC MAIT cells with PMA/ionomycin for 5 hours, showed an increased production of IL-8 (117), a cytokine that promotes tumor progression and angiogenesis. MAIT cells, normally, are able to kill target cells (118), but in HCC, their ability to produce granzyme B and perforin is decreased (117). Infiltration of MAIT cells in HCC is reduced due to the lower expression of the chemokine receptors, CXCR6, CCR6, and CCR9 (117). The reduced presence of MAIT cells in the tumor microenvironment is, also, associated with apoptosis. Single-cell analysis from HCC samples revealed that MAIT cells express genes related to apoptotic pathways (114). Therefore, the number of MAIT cells is reduced and the remaining MAIT cells are dysfunctional in HCC (**Figure 3**).

#### 3.3.2 iNKT cells in HCC

iNKT cell number is reduced in late stages of HCC (stages III, IV) in tissue (119) and blood samples (120, 121) from HCC patients. Expression of inhibitory receptors in iNKT cells, such as TIM3, CTLA4, PDL1, is increased and PD1^+^ iNKT cells produce lower amounts of IFNγ showing an exhausted phenotype and impaired function (121, 122). This impaired function may happen due to chronic TCR stimulus; Indeed, there is a recent study showing that long-chain Acylcarnitines (LCACs) derived from tumor cells can lead to impaired function of iNKT cells through TCR signalling (122). Blocking the PD1/PDL1 pathway using anti-PD1 blockade improved production of cytokines (IFNγ, TNFα) from iNKT cells after PMA/ionomycin stimulation (121). Patients with increased presence of iNKT cells had a better overall survival (OS) compared to those with lower numbers of iNKT cells (123, 124). Radiotherapy in patients with HCC, increased the number of iNKT cells after 3 months and patients with increased iNKT cell number achieved higher 2-year OS (125). Adoptive transfer of *in vitro* expanded autologous iNKT cells in patients with HCC reduced the expression level of the HCC marker a-fetoprotein; in addition, both overall and progression-free survival increased in four out of ten patients, while one patient survived without tumor recurrence, indicating that therapy using iNKT cells is promising and safe and may be combined with other therapies against HCC (126).

In mouse cancer models, iNKT cells promote liver damage, although the mechanisms related to HCC development are not clear (127). Murine studies using an orthotopic HCC mouse model showed that activation of iNKT cells with aGalCer suppressed tumor development, while the NK cell cytotoxic activity increased (128). In the transgenic L-type pyruvate kinase Lpk-myc^+^ HCC mouse model, depletion of iNKT cells using anti-NK1.1 accelerated progression of liver tumors induced by β-catenin (129). Adoptive transfer of *ex vivo* activated iNKT cells suppressed HCC development in mice (130). In *Fkbp5^−/−^* mice treated with DEN, progression of HCC was inhibited and T cells, including iNKT cells, were significantly increased compared to *WT* (131). Hepatic iNKT, NK, and T cells express CXCR6 (83, 132). *Cxcr6^−/−^* mice, which are characterized by a reduced number of hepatic iNKT cells, treated short-term with CCL_4_ or fed with MCD diet were protected from fibrosis (83)., while adoptive transfer of iNKT cells but conventional CD4^+^ T cells enhanced fibrogenesis (83). However, *Cxcr6^−/−^* mice treated with DEN developed more tumors than *WT* mice, while the number of iNKT cells and their ability to produce IFNγ was decreased (133). Adoptive transfer of CD4^+^ T or iNKT cells in *Cxcr6^−/−^* DEN mice reduced the number of senescent cells, indicating that during HCC development, CD4^+^ and iNKT cells participate in the surveillance of senescent cells (133). Surveillance of senescent cells is associated with increased amounts of IFNγ and TNFα produced by iNKT cells (134, 135). Treatment with aGalCer reduced the number of senescent hepatocytes (133). Importantly, HCC cells produced TGFβ and suppressed the anti-oncogenic functions of iNKT cells and T cells (136). In contrast, in CD-HFD fed mice, iNKT cells contributed to NASH and HCC development through production of Light and enhanced lipid uptake from hepatocytes. *Light^−/−^* mice were protected from NASH and HCC with no increase in ALT and cholesterol levels and no change in the number and activation of CD8^+^ and iNKT cells (98). Importantly, CD-HFD causes severe steatosis and fibrosis before HCC, whereas DEN-mediated HCC does not involve fibrogenesis. Taken together, these results indicate that iNKT cells contribute to HCC tumorigenesis through promotion of hepatic fibrosis; however, iNKT cells may have anti-oncogenic functions in established HCC tumors (**Figure 3**).

## 4 Conclusions

The liver is enriched in tissue-resident innate-like T cells, including NKT and MAIT cells, thus suggesting that these cellular types may be potentially important for the hepatic immunity. Importantly, the distinct ability of NKT and MAIT cells to recognize non-peptide antigens, such as lipids and microbial metabolites, through their TCRs renders them potential early sensors of altered liver function; in addition, they respond early during viral infection through TCR-independent mechanisms of activation, thus potentially serving as early biomarkers and predictors of hepatic diseases. However, understanding how they operate during chronic liver diseases is intrinsically complicated, because their functions change depending on the stage of the disease and the mode and degree of their activation. As a consequence, the range of the cytokines and chemokines secreted by innate-like T cells is skewed, thus influencing the function of bystander immune cells. Therefore, delineation of the molecular interactions involved in the cross-talk between innate-like T cells and the surrounding immune cell microenvironment will contribute to understanding how hepatic immune tolerance is maintained. This is particularly important, because current immune cell-based therapies focusing on NKT cells in humans have shown promising results; however, most of the current knowledge on NKT and MAIT cell functions comes mainly from studies in mouse models, which may not reliably recapitulate the human condition. Therefore, additional clinical studies will shed light on the potential therapeutic applications of innate-like T cells against liver diseases. The availability of reagents that unambiguously identify these unique immunological subsets and distinguish them from other lymphocytes enables specific monitoring of their functions during hepatic homeostasis and disease.

## Author contributions

MP drafted the manuscript, MV supervised and drafted the manuscript. All authors contributed to the article and approved the submitted version.

## References

[1] Trefts E Gannon M Wasserman DH. The liver. Curr Biol (2017) 27(21):R1147–51. doi: 10.1016/j.cub.2017.09.019 PMC5897118 29112863

[2] Chen D Le TH Shahidipour H Read SA Ahlenstiel G. The role of gut-derived microbial antigens on liver fibrosis initiation and progression. Cells (2019) 8(11). doi: 10.3390/cells8111324 PMC6912265 31717860

[3] Ficht X Iannacone M. Immune surveillance of the liver by T cells. Sci Immunol (2020) 5(51). doi: 10.1126/sciimmunol.aba2351 32887842

[4] Heymann F Tacke F. Immunology in the liver–from homeostasis to disease. Nat Rev Gastroenterol Hepatol (2016) 13(2):88–110. doi: 10.1038/nrgastro.2015.200 26758786

[5] Verykokakis M Zook EC Kee BL. ID'ing Innate and innate-like lymphoid cells. Immunol Rev (2014) 261(1):177–97. doi: 10.1111/imr.12203 PMC4159719 25123285

[6] Bendelac A Savage PB Teyton L. The biology of NKT cells. Annu Rev Immunol (2007) 25:297–336. doi: 10.1146/annurev.immunol.25.022106.141711 17150027

[7] Egawa T Eberl G Taniuchi I Benlagha K Geissmann F Hennighausen L. Genetic evidence supporting selection of the Valpha14i NKT cell lineage from double-positive thymocyte precursors. Immunity (2005) 22(6):705–16. doi: 10.1016/j.immuni.2005.03.011 15963785

[8] Gapin L Matsuda JL Surh CD Kronenberg M. NKT cells derive from double-positive thymocytes that are positively selected by CD1d. Nat Immunol (2001) 2(10):971–8. doi: 10.1038/ni710 11550008

[9] Kawano T Cui J Koezuka Y Toura I Kaneko Y Motoki K. CD1d-restricted and TCR-mediated activation of valpha14 NKT cells by glycosylceramides. Science (1997) 278(5343):1626–9. doi: 10.1126/science.278.5343.1626 9374463

[10] Brennan PJ Brigl M Brenner MB. Invariant natural killer T cells: An innate activation scheme linked to diverse effector functions. Nat Rev Immunol (2013) 13(2):101–17. doi: 10.1038/nri3369 23334244

[11] Coquet JM Chakravarti S Kyparissoudis K McNab FW Pitt LA McKenzie BS. Diverse cytokine production by NKT cell subsets and identification of an IL-17-producing CD4-NK1.1- NKT cell population. Proc Natl Acad Sci USA (2008) 105(32):11287–92. doi: 10.1073/pnas.0801631105 PMC2516267 18685112

[12] Singh AK Tripathi P Cardell SL. Type II NKT cells: An elusive population with immunoregulatory properties. Front Immunol (2018) 9:1969. doi: 10.3389/fimmu.2018.01969 30210505 PMC6120993

[13] Matsuda JL Naidenko OV Gapin L Nakayama T Taniguchi M Wang CR. Tracking the response of natural killer T cells to a glycolipid antigen using CD1d tetramers. J Exp Med (2000) 192(5):741–54. doi: 10.1084/jem.192.5.741 PMC2193268 10974039

[14] Benlagha K Weiss A Beavis A Teyton L Bendelac A. *In vivo* identification of glycolipid antigen-specific T cells using fluorescent CD1d tetramers. J Exp Med (2000) 191(11):1895–903. doi: 10.1084/jem.191.11.1895 PMC2213523 10839805

[15] Ambrosino E Terabe M Halder RC Peng J Takaku S Miyake S. Cross-regulation between type I and type II NKT cells in regulating tumor immunity: A new immunoregulatory axis. J Immunol (2007) 179(8):5126–36. doi: 10.4049/jimmunol.179.8.5126 17911598

[16] Lee YJ Starrett GJ Lee ST Yang R Henzler CM Jameson SC. Lineage-specific effector signatures of invariant NKT cells are shared amongst γδ T, innate lymphoid, and Th cells. J Immunol (2016) 197(4):1460–70. doi: 10.4049/jimmunol.1600643 PMC4976040 27385777

[17] Griewank K Borowski C Rietdijk S Wang N Julien A Wei DG. Homotypic interactions mediated by Slamf1 and Slamf6 receptors control NKT cell lineage development. Immunity (2007) 27(5):751–62. doi: 10.1016/j.immuni.2007.08.020 PMC2170879 18031695

[18] Savage AK Constantinides MG Han J Picard D Martin E Li B. The transcription factor PLZF directs the effector program of the NKT cell lineage. Immunity (2008) 29(3):391–403. doi: 10.1016/j.immuni.2008.07.011 18703361 PMC2613001

[19] Kovalovsky D Uche OU Eladad S Hobbs RM Yi W Alonzo E. The BTB-zinc finger transcriptional regulator PLZF controls the development of invariant natural killer T cell effector functions. Nat Immunol (2008) 9(9):1055–64. doi: 10.1038/ni.1641 PMC2662733 18660811

[20] Lee YJ Holzapfel KL Zhu J Jameson SC Hogquist KA. Steady-state production of IL-4 modulates immunity in mouse strains and is determined by lineage diversity of iNKT cells. Nat Immunol (2013) 14(11):1146–54. doi: 10.1038/ni.2731 PMC3824254 24097110

[21] Koay HF Gherardin NA Enders A Loh L Mackay LK Almeida CF. A three-stage intrathymic development pathway for the mucosal-associated invariant T cell lineage. Nat Immunol (2016) 17(11):1300–11. doi: 10.1038/ni.3565 27668799

[22] Seach N Guerri L Le Bourhis L Mburu Y Cui Y Bessoles S. Double-positive thymocytes select mucosal-associated invariant T cells. J Immunol (2013) 191(12):6002–9. doi: 10.4049/jimmunol.1301212 24244014

[23] Tilloy F Treiner E Park SH Garcia C Lemonnier F De La Salle H. An invariant T cell receptor alpha chain defines a novel TAP-independent major histocompatibility complex class ib-restricted alpha/beta T cell subpopulation in mammals. J Exp Med (1999) 189(12):1907–21. doi: 10.1084/jem.189.12.1907 PMC2192962 10377186

[24] Rahimpour A Koay HF Enders A Clanchy R Eckle SBG Meehan B. Identification of phenotypically and functionally heterogeneous mouse mucosal-associated invariant T cells using MR1 tetramers. J Exp Med (2015) 212(7):1095–108. doi: 10.1084/jem.20142110 PMC4493408 26101265

[25] Treiner E Duban L Bahram S Radosavljevic M Wanner V Tilloy F. Selection of evolutionarily conserved mucosal-associated invariant T cells by MR1. Nature (2003) 422(6928):164–9. doi: 10.1038/nature01433 12634786

[26] Kjer-Nielsen L Patel O Corbett AJ Le Nours J Meehan B Liu L. MR1 presents microbial vitamin b metabolites to MAIT cells. Nature (2012) 491(7426):717–23. doi: 10.1038/nature11605 23051753

[27] Reantragoon R Corbett AJ Sakala IG Gherardin NA Furness JB Chen Z. Antigen-loaded MR1 tetramers define T cell receptor heterogeneity in mucosal-associated invariant T cells. J Exp Med (2013) 210(11):2305–20. doi: 10.1084/jem.20130958 PMC3804952 24101382

[28] Huang W He W Shi X He X Dou L Gao Y. The role of CD1d and MR1 restricted T cells in the liver. Front Immunol (2018) 9:2424. doi: 10.3389/fimmu.2018.02424 30425710 PMC6218621

[29] Elizalde MM Tadey L Mammana L Quarleri JF Campos RH Flichman DM. Biological characterization of hepatitis b virus genotypes: Their role in viral replication and antigen expression. Front Microbiol (2021) 12:758613. doi: 10.3389/fmicb.2021.758613 34803982 PMC8600256

[30] Leumi S Bigna JJ Amougou MA Ngouo A Nyaga UF Noubiap JJ. Global burden of hepatitis b infection in people living with human immunodeficiency virus: A systematic review and meta-analysis. Clin Infect Dis (2020) 71(11):2799–806. doi: 10.1093/cid/ciz1170 31813969

[31] Tang LSY Covert E Wilson E Kottilil S. Chronic hepatitis b infection: A review. JAMA (2018) 319(17):1802–13. doi: 10.1001/jama.2018.3795 29715359

[32] Wilkins T Zimmerman D Schade RR. Hepatitis b: diagnosis and treatment. Am Fam Physician (2010) 81(8):965–72. 20387772

[33] Manns MP Buti M Gane E Pawlotsky JM Razavi H Terrault N. Hepatitis c virus infection. Nat Rev Dis Primers (2017) 3:17006. doi: 10.1038/nrdp.2017.6 28252637

[34] Puchades Renau L Berenguer M. Introduction to hepatitis c virus infection: Overview and history of hepatitis c virus therapies. Hemodial Int (2018) 22 Suppl 1:S8–S21. doi: 10.1111/hdi.12647 29694724

[35] EAftSotLEa. EASL recommendations on treatment of hepatitis c 2016. J Hepatol (2017) 66(1):153–94. doi: 10.1016/j.jhep.2016.09.001 27667367

[36] Sarrazin C. Treatment failure with DAA therapy: Importance of resistance. J Hepatol (2021) 74(6):1472–82. doi: 10.1016/j.jhep.2021.03.004 33716089

[37] Huang W He W Shi X Ye Q He X Dou L. Mucosal-associated invariant T-cells are severely reduced and exhausted in humans with chronic HBV infection. J Viral Hepat (2020) 27(11):1096–107. doi: 10.1111/jvh.13341 32510704

[38] Yong YK Tan HY Saeidi A Rosmawati M Atiya N Ansari AW. Decrease of CD69 levels on TCR Vα7.2+ CD4^+^ inn^a^te-like lymphocytes is associated with impaired cytotoxic functions in chronic hepatitis b virus-infected patients. Innate Immun (2017) 23(5):459–67. doi: 10.1177/1753425917714854 28606013

[39] Yong YK Saeidi A Tan HY Rosmawati M Enström PF Al Batran R. Hyper-expression of PD-1 is associated with the levels of exhausted and dysfunctional phenotypes of irculating CD161++ TC^R^ iVα7.2+ mu^cos^al-associated invariant T cells in chronic hepatits b virus infection. Front Immunol (2018) 9:472. doi: 10.3389/fimmu.2018.00472 29616020 PMC5868455

[40] Xue H Li H Ju LL Han XF Cheng TC Luo X. Mucosal-associated invariant T cells in hepatitis b virus-related liver failure. World J Gastroenterol (2020) 26(31):4703–17. doi: 10.3748/wjg.v26.i31.4703 PMC7445862 32884227

[41] Liu Y Zhu P Wang W Tan X Liu C Chen Y. Mucosal-associated invariant T cell dysregulation correlates with conjugated bilirubin level in chronic HBV infection. Hepatology (2021) 73(5):1671–87. doi: 10.1002/hep.31602 33080074

[42] Boeijen LL Montanari NR de Groen RA Van Oord GW Van der Heide-Mulder M De Knegt RJ. Mucosal-associated invariant T cells are more activated in chronic hepatitis b, but not depleted in blood: Reversal by antiviral therapy. J Infect Dis (2017) 216(8):969–76. doi: 10.1093/infdis/jix425 28968772

[43] van Wilgenburg B Scherwitzl I Hutchinson EC Leng T Kurioka A Kulicke C. MAIT cells are activated during human viral infections. Nat Commun (2016) 7:11653. doi: 10.1038/ncomms11653 27337592 PMC4931007

[44] Hengst J Strunz B Deterding K Ljunggren HG Leeansyah E Manns MP. Nonreversible MAIT cell-dysfunction in chronic hepatitis c virus infection despite successful interferon-free therapy. Eur J Immunol (2016) 46(9):2204–10. doi: 10.1002/eji.201646447 27296288

[45] Barathan M Mohamed R Vadivelu J Chang LY Saeidi A Yong YK. Peripheral loss of CD8(+) CD161(++) TCRVα7·2(+) mucosal-associated invariant T cells in chronic hepatitis c virus-infected patients. Eur J Clin Invest (2016) 46(2):170–80. doi: 10.1111/eci.12581 26681320

[46] Bolte FJ O'Keefe AC Webb LM Serti E Rivera E Liang TJ. Intra-hepatic depletion of mucosal-associated invariant T cells in hepatitis c virus-induced liver inflammation. Gastroenterology (2017) 153(5):1392–1403.e2. doi: 10.1053/j.gastro.2017.07.043 28780074 PMC5669813

[47] Du Y Khera T Strunz B Deterding K Todt D Woller N. Imprint of unconventional T-cell response in acute hepatitis c persists despite successful early antiviral treatment. Eur J Immunol (2022) 52(3):472–83. doi: 10.1002/eji.202149457 34843107

[48] Khlaiphuengsin A Chuaypen N Sodsai P Reantragoon R Han WM Avihingsanon A. Successful direct-acting antiviral therapy improves circulating mucosal-associated invariant T cells in patients with chronic HCV infection. PloS One (2020) 15(12):e0244112. doi: 10.1371/journal.pone.0244112 33382729 PMC7775079

[49] Beudeker BJB van Oord GW Arends JE Zur Wiesch JS van der Heide MS de Knegt RJ. Mucosal-associated invariant T-cell frequency and function in blood and liver of HCV mono- and HCV/HIV co-infected patients with advanced fibrosis. Liver Int (2018) 38(3):458–68. doi: 10.1111/liv.13544 PMC5836956 28792648

[50] Spaan M van Oord G Kreefft K Hou J Hansen BE Janssen HLA. Immunological analysis during interferon-free therapy for chronic hepatitis c virus infection reveals modulation of the natural killer cell compartment. J Infect Dis (2016) 213(2):216–23. doi: 10.1093/infdis/jiv391 26223768

[51] Zeissig S Murata K Sweet L Publicover J Hu Z Kaser A. Hepatitis b virus-induced lipid alterations contribute to natural killer T cell-dependent protective immunity. Nat Med (2012) 18(7):1060–8. doi: 10.1038/nm.2811 PMC3478098 22706385

[52] Ito H Ando K Ishikawa T Nakayama T Taniguchi M Saito K. Role of Valpha14+ NKT cells in the development of hepatitis b virus-specific CTL: activation of Valpha14+ NKT cells promotes the breakage of CTL tolerance. Int Immunol (2008) 20(7):869–79. doi: 10.1093/intimm/dxn046 18487227

[53] Kakimi K Guidotti LG Koezuka Y Chisari FV. Natural killer T cell activation inhibits hepatitis b virus replication in vivo. J Exp Med (2000) 192(7):921–30. doi: 10.1084/jem.192.7.921 PMC2193313 11015434

[54] Wang XF Lei Y Chen M Chen CB Ren H Shi TD. PD-1/PDL1 and CD28/CD80 pathways modulate natural killer T cell function to inhibit hepatitis b virus replication. J Viral Hepat (2013) 20 Suppl 1:27–39. doi: 10.1111/jvh.12061 23458522

[55] Yang Z Lei Y Chen C Ren H Shi T. Roles of the programmed cell death 1, T cell immunoglobulin mucin-3, and cluster of differentiation 288 pathways in the low reactivity of invariant natural killer T cells after chronic hepatitis b virus infection. Arch Virol (2015) 160(10):2535–45. doi: 10.1007/s00705-015-2539-3 26215444

[56] Xu Y Wang Z Du X Liu Y Song X Wang T. Tim-3 blockade promotes iNKT cell function to inhibit HBV replication. J Cell Mol Med (2018) 22(6):3192–201. doi: 10.1111/jcmm.13600 PMC5980221 29602251

[57] Wei X Qian J Yao W Chen L Guan H Chen Y. Hyperactivated peripheral invariant natural killer T cells correlate with the progression of HBV-relative liver cirrhosis. Scand J Immunol (2019) 90(2):e12775. doi: 10.1111/sji.12775 31069827

[58] Jin Z Sun R Wei H Gao X Chen Y Tian Z. Accelerated liver fibrosis in hepatitis b virus transgenic mice: Involvement of natural killer T cells. Hepatology (2011) 53(1):219–29. doi: 10.1002/hep.23983 21140473

[59] Lucas M Gadola S Meier U Young NT Harcourt G Karadimitris A. Frequency and phenotype of circulating Valpha24/Vbeta11 double-positive natural killer T cells during hepatitis c virus infection. J Virol (2003) 77(3):2251–7. doi: 10.1128/jvi.77.3.2251-2257.2003 PMC140901 12525661

[60] Yamagiwa S Matsuda Y Ichida T Honda Y Takamura M Sugahara S. Sustained response to interferon-alpha plus ribavirin therapy for chronic hepatitis c is closely associated with increased dynamism of intrahepatic natural killer and natural killer T cells. Hepatol Res (2008) 38(7):664–72. doi: 10.1111/j.1872-034X.2008.00317.x 18328072

[61] de Lalla C Galli G Aldrighetti L Romeo R Mariani M Monno A. Production of profibrotic cytokines by invariant NKT cells characterizes cirrhosis progression in chronic viral hepatitis. J Immunol (2004) 173(2):1417–25. doi: 10.4049/jimmunol.173.2.1417 15240738

[62] Miyaki E Hiraga N Imamura M Uchida T Kan H Tsuge M. Interferon alpha treatment stimulates interferon gamma expression in type I NKT cells and enhances their antiviral effect against hepatitis c virus. PloS One (2017) 12(3):e0172412. doi: 10.1371/journal.pone.0172412 28253324 PMC5333814

[63] Smith BW Adams LA. Non-alcoholic fatty liver disease. Crit Rev Clin Lab Sci (2011) 48(3):97–113. doi: 10.3109/10408363.2011.596521 21875310

[64] Pafili K Roden M. Nonalcoholic fatty liver disease (NAFLD) from pathogenesis to treatment concepts in humans. Mol Metab (2021) 50:101122. doi: 10.1016/j.molmet.2020.101122 33220492 PMC8324683

[65] Wang H Mehal W Nagy LE Rotman Y. Immunological mechanisms and therapeutic targets of fatty liver diseases. Cell Mol Immunol (2021) 18(1):73–91. doi: 10.1038/s41423-020-00579-3 33268887 PMC7852578

[66] Wan J Benkdane M Teixeira-Clerc F Bonnafous S Louvet A Lafdil F. M2 kupffer cells promote M1 kupffer cell apoptosis: A protective mechanism against alcoholic and nonalcoholic fatty liver disease. Hepatology (2014) 59(1):130–42. doi: 10.1002/hep.26607 23832548

[67] Nd AM. Non-alcoholic fatty liver disease, an overview. Integr Med (Encinitas) (2019) 18(2):42–9. PMC6601444 31341444

[68] Huby T Gautier EL. Immune cell-mediated features of non-alcoholic steatohepatitis. Nat Rev Immunol (2022) 22(7):429–43. doi: 10.1038/s41577-021-00639-3 PMC8570243 34741169

[69] Marchesini G Marzocchi R. Metabolic syndrome and NASH. Clin Liver Dis (2007) 11(1):105–17. doi: 10.1016/j.cld.2007.02.013 17544974

[70] Friedman SL Neuschwander-Tetri BA Rinella M Sanyal AJ. Mechanisms of NAFLD development and therapeutic strategies. Nat Med (2018) 24(7):908–22. doi: 10.1038/s41591-018-0104-9 PMC6553468 29967350

[71] Anstee QM Goldin RD. Mouse models in non-alcoholic fatty liver disease and steatohepatitis research. Int J Exp Pathol (2006) 87(1):1–16. doi: 10.1111/j.0959-9673.2006.00465.x 16436109 PMC2517349

[72] Kulinski A Vance DE Vance JE. A choline-deficient diet in mice inhibits neither the CDP-choline pathway for phosphatidylcholine synthesis in hepatocytes nor apolipoprotein b secretion. J Biol Chem (2004) 279(23):23916–24. doi: 10.1074/jbc.M312676200 15024002

[73] Raubenheimer PJ Nyirenda MJ Walker BR. A choline-deficient diet exacerbates fatty liver but attenuates insulin resistance and glucose intolerance in mice fed a high-fat diet. Diabetes (2006) 55(7):2015–20. doi: 10.2337/db06-0097 16804070

[74] Recena Aydos L Aparecida do Amaral L Serafim de Souza R Jacobowski AC Freitas Dos Santos E Rodrigues Macedo ML. Nonalcoholic fatty liver disease induced by high-fat diet in C57bl/6 models. Nutrients (2019) 11(12). doi: 10.3390/nu11123067 PMC6949901 31888190

[75] White PA Cercato LM Araújo JM Souza LA Soares AF Barbosa AP. Model of high-fat diet-induced obesity associated to insulin resistance and glucose intolerance. Arq Bras Endocrinol Metabol (2013) 57(5):339–45. doi: 10.1590/s0004-27302013000500002 23896799

[76] Nakagawa H. Recent advances in mouse models of obesity- and nonalcoholic steatohepatitis-associated hepatocarcinogenesis. World J Hepatol (2015) 7(17):2110–8. doi: 10.4254/wjh.v7.i17.2110 PMC4539404 26301053

[77] Xin X Cai BY Chen C Tian HJ Wang X Hu YY. High-trans fatty acid and high-sugar diets can cause mice with non-alcoholic steatohepatitis with liver fibrosis and potential pathogenesis. Nutr Metab (Lond) (2020) 17:40. doi: 10.1186/s12986-020-00462-y 32508961 PMC7249374

[78] Scholten D Trebicka J Liedtke C Weiskirchen R. The carbon tetrachloride model in mice. Lab Anim. (2015) 49(1 Suppl):4–11. doi: 10.1177/0023677215571192 25835733

[79] Godfrey DI Koay HF McCluskey J Gherardin NA. The biology and functional importance of MAIT cells. Nat Immunol (2019) 20(9):1110–28. doi: 10.1038/s41590-019-0444-8 31406380

[80] Li Y Huang B Jiang X Chen W Zhang J Wei Y. Mucosal-associated invariant T cells improve nonalcoholic fatty liver disease through regulating macrophage polarization. Front Immunol (2018) 9:1994. doi: 10.3389/fimmu.2018.01994 30233587 PMC6131560

[81] Hegde P Weiss E Paradis V Wan J Mabire M Sukriti S. Mucosal-associated invariant T cells are a profibrogenic immune cell population in the liver. Nat Commun (2018) 9(1):2146. doi: 10.1038/s41467-018-04450-y 29858567 PMC5984626

[82] Diedrich T Kummer S Galante A Drolz A Schlicker V Lohse AW. Characterization of the immune cell landscape of patients with NAFLD. PloS One (2020) 15(3):e0230307. doi: 10.1371/journal.pone.0230307 32168345 PMC7069622

[83] Wehr A Baeck C Heymann F Niemietz PM Hammerich L Martin C. Chemokine receptor CXCR6-dependent hepatic NK T cell accumulation promotes inflammation and liver fibrosis. J Immunol (2013) 190(10):5226–36. doi: 10.4049/jimmunol.1202909 23596313

[84] Kremer M Thomas E Milton RJ Perry AW van Rooijen N Wheeler MD. Kupffer cell and interleukin-12-dependent loss of natural killer T cells in hepatosteatosis. Hepatology (2010) 51(1):130–41. doi: 10.1002/hep.23292 PMC3761962 20034047

[85] Zhu H Zhang Q Chen G. CXCR6 deficiency ameliorates ischemia-reperfusion injury by reducing the recruitment and cytokine production of hepatic NKT cells in a mouse model of non-alcoholic fatty liver disease. Int Immunopharmacol (2019) 72:224–34. doi: 10.1016/j.intimp.2019.04.021 31002999

[86] Tiemessen MM Jagger AL Evans HG van Herwijnen MJ John S Taams LS. CD4+CD25+Foxp3+ regulatory T cells induce alternative activation of human monocytes/macrophages. Proc Natl Acad Sci USA (2007) 104(49):19446–51. doi: 10.1073/pnas.0706832104 PMC2148309 18042719

[87] Tajiri K Shimizu Y Tsuneyama K Sugiyama T. Role of liver-infiltrating CD3+CD56+ natural killer T cells in the pathogenesis of nonalcoholic fatty liver disease. Eur J Gastroenterol Hepatol (2009) 21(6):673–80. doi: 10.1097/MEG.0b013e32831bc3d6 19318971

[88] Syn WK Oo YH Pereira TA Karaca GF Jung Y Omenetti A. Accumulation of natural killer T cells in progressive nonalcoholic fatty liver disease. Hepatology (2010) 51(6):1998–2007. doi: 10.1002/hep.23599 20512988 PMC2920131

[89] Adler M Taylor S Okebugwu K Yee H Fielding C Fielding G. Intrahepatic natural killer T cell populations are increased in human hepatic steatosis. World J Gastroenterol (2011) 17(13):1725–31. doi: 10.3748/wjg.v17.i13.1725 PMC3072637 21483633

[90] Li Z Lin H Yang S Diehl AM. Murine leptin deficiency alters kupffer cell production of cytokines that regulate the innate immune system. Gastroenterology (2002) 123(4):1304–10. doi: 10.1053/gast.2002.35997 12360490

[91] Elinav E Pappo O Sklair-Levy M Margalit M Shibolet O Gomori M. Adoptive transfer of regulatory NKT lymphocytes ameliorates non-alcoholic steatohepatitis and glucose intolerance in ob/ob mice and is associated with intrahepatic CD8 trapping. J Pathol (2006) 209(1):121–8. doi: 10.1002/path.1950 16482497

[92] Li Z Soloski MJ Diehl AM. Dietary factors alter hepatic innate immune system in mice with nonalcoholic fatty liver disease. Hepatology (2005) 42(4):880–5. doi: 10.1002/hep.20826 16175608

[93] Tang ZH Liang S Potter J Jiang X Mao HQ Li Z. Tim-3/galectin-9 regulate the homeostasis of hepatic NKT cells in a murine model of nonalcoholic fatty liver disease. J Immunol (2013) 190(4):1788–96. doi: 10.4049/jimmunol.1202814 PMC3563933 23296703

[94] Sutti S Jindal A Locatelli I Vacchiano M Gigliotti L Bozzola C. Adaptive immune responses triggered by oxidative stress contribute to hepatic inflammation in NASH. Hepatology (2014) 59(3):886–97. doi: 10.1002/hep.26749 24115128

[95] Locatelli I Sutti S Vacchiano M Bozzola C Albano E. NF-κB1 deficiency stimulates the progression of non-alcoholic steatohepatitis (NASH) in mice by promoting NKT-cell-mediated responses. Clin Sci (Lond) (2013) 124(4):279–87. doi: 10.1042/CS20120289 22970906

[96] Syn WK Agboola KM Swiderska M Michelotti GA Liaskou E Pang H. NKT-associated hedgehog and osteopontin drive fibrogenesis in non-alcoholic fatty liver disease. Gut (2012) 61(9):1323–9. doi: 10.1136/gutjnl-2011-301857 PMC3578424 22427237

[97] Syn WK Choi SS Liaskou E Karaca GF Agboola KM Oo YH. Osteopontin is induced by hedgehog pathway activation and promotes fibrosis progression in nonalcoholic steatohepatitis. Hepatology (2011) 53(1):106–15. doi: 10.1002/hep.23998 PMC3025083 20967826

[98] Wolf MJ Adili A Piotrowitz K Abdullah Z Boege Y Stemmer K. Metabolic activation of intrahepatic CD8+ T cells and NKT cells causes nonalcoholic steatohepatitis and liver cancer *via* cross-talk with hepatocytes. Cancer Cell (2014) 26(4):549–64. doi: 10.1016/j.ccell.2014.09.003 25314080

[99] Bhattacharjee J Kirby M Softic S Miles L Salazar-Gonzalez RM Shivakumar P. Hepatic natural killer T-cell and CD8+ T-cell signatures in mice with nonalcoholic steatohepatitis. Hepatol Commun (2017) 1(4):299–310. doi: 10.1002/hep4.1041 29152605 PMC5687094

[100] Siegel RL Miller KD Fuchs HE Jemal A. Cancer statistics, 2022. CA Cancer J Clin (2022) 72(1):7–33. doi: 10.3322/caac.21708 35020204

[101] Tsuchiya N Sawada Y Endo I Saito K Uemura Y Nakatsura T. Biomarkers for the early diagnosis of hepatocellular carcinoma. World J Gastroenterol (2015) 21(37):10573–83. doi: 10.3748/wjg.v21.i37.10573 PMC4588079 26457017

[102] El-Serag HB Rudolph KL. Hepatocellular carcinoma: epidemiology and molecular carcinogenesis. Gastroenterology (2007) 132(7):2557–76. doi: 10.1053/j.gastro.2007.04.061 17570226

[103] Yang JD Hainaut P Gores GJ Amadou A Plymoth A Roberts LR. A global view of hepatocellular carcinoma: trends, risk, prevention and management. Nat Rev Gastroenterol Hepatol (2019) 16(10):589–604. doi: 10.1038/s41575-019-0186-y 31439937 PMC6813818

[104] Fu J Xu D Liu Z Shi M Zhao P Fu B. Increased regulatory T cells correlate with CD8 T-cell impairment and poor survival in hepatocellular carcinoma patients. Gastroenterology (2007) 132(7):2328–39. doi: 10.1053/j.gastro.2007.03.102 17570208

[105] Matsui M Machida S Itani-Yohda T Akatsuka T. Downregulation of the proteasome subunits, transporter, and antigen presentation in hepatocellular carcinoma, and their restoration by interferon-gamma. J Gastroenterol Hepatol (2002) 17(8):897–907. doi: 10.1046/j.1440-1746.2002.02837.x 12164966

[106] Ninomiya T Akbar SM Masumoto T Horiike N Onji M. Dendritic cells with immature phenotype and defective function in the peripheral blood from patients with hepatocellular carcinoma. J Hepatol (1999) 31(2):323–31. doi: 10.1016/s0168-8278(99)80231-1 10453947

[107] Wu K Kryczek I Chen L Zou W Welling TH. Kupffer cell suppression of CD8+ T cells in human hepatocellular carcinoma is mediated by B7-H1/programmed death-1 interactions. Cancer Res (2009) 69(20):8067–75. doi: 10.1158/0008-5472.CAN-09-0901 PMC4397483 19826049

[108] Hoechst B Ormandy LA Ballmaier M Lehner F Krüger C Manns MP. A new population of myeloid-derived suppressor cells in hepatocellular carcinoma patients induces CD4(+)CD25(+)Foxp3(+) T cells. Gastroenterology (2008) 135(1):234–43. doi: 10.1053/j.gastro.2008.03.020 18485901

[109] Lu LC Chang CJ Hsu CH. Targeting myeloid-derived suppressor cells in the treatment of hepatocellular carcinoma: Current state and future perspectives. J Hepatocell Carcinoma (2019) 6:71–84. doi: 10.2147/JHC.S159693 31123667 PMC6511249

[110] Ghiringhelli F Ménard C Martin F Zitvogel L. The role of regulatory T cells in the control of natural killer cells: relevance during tumor progression. Immunol Rev (2006) 214:229–38. doi: 10.1111/j.1600-065X.2006.00445.x 17100888

[111] Zahran AM Abdel-Meguid MM Ashmawy AM Rayan A Elkady A Elsherbiny NM. Frequency and implications of natural killer and natural killer T cells in hepatocellular carcinoma. Egypt J Immunol (2018) 25(2):45–52. 30600947

[112] Dusseaux M Martin E Serriari N Péguillet I Premel V Louis D. Human MAIT cells are xenobiotic-resistant, tissue-targeted, CD161hi IL-17-secreting T cells. Blood (2011) 117(4):1250–9. doi: 10.1182/blood-2010-08-303339 21084709

[113] Zheng C Zheng L Yoo JK Guo H Zhang Y Guo X. Landscape of infiltrating T cells in liver cancer revealed by single-cell sequencing. Cell (2017) 169(7):1342–1356.e16. doi: 10.1016/j.cell.2017.05.035 28622514

[114] Huang W Ye D He W He X Shi X Gao Y. Activated but impaired IFN-γ production of mucosal-associated invariant T cells in patients with hepatocellular carcinoma. J Immunother Cancer (2021) 119(11). doi: 10.1136/jitc-2021-003685 PMC8601081 34789552

[115] Ussher JE Bilton M Attwod E Shadwell J Richardson R de Lara C. CD161++ CD8+ T cells, including the MAIT cell subset, are specifically activated by IL-12+IL-18 in a TCR-independent manner. Eur J Immunol (2014) 44(1):195–203. doi: 10.1002/eji.201343509 24019201 PMC3947164

[116] Jeffery HC van Wilgenburg B Kurioka A Parekh K Stirling K Roberts S. Biliary epithelium and liver b cells exposed to bacteria activate intrahepatic MAIT cells through MR1. J Hepatol (2016) 64(5):1118–27. doi: 10.1016/j.jhep.2015.12.017 PMC4822535 26743076

[117] Duan M Goswami S Shi JY Wu LJ Wang XY Ma JQ. Activated and exhausted MAIT cells foster disease progression and indicate poor outcome in hepatocellular carcinoma. Clin Cancer Res (2019) 25(11):3304–16. doi: 10.1158/1078-0432.CCR-18-3040 30723143

[118] Kurioka A Walker LJ Klenerman P Willberg CB. MAIT cells: New guardians of the liver. Clin Transl Immunol (2016) 5(8):e98. doi: 10.1038/cti.2016.51 PMC5007630 27588203

[119] Li X Peng J Pang Y Yu S Yu X Chen P. Identification of a FOXP3(+)CD3(+)CD56(+) population with immunosuppressive function in cancer tissues of human hepatocellular carcinoma. Sci Rep (2015) 5:14757. doi: 10.1038/srep14757 26437631 PMC4594002

[120] Bricard G Cesson V Devevre E Bouzourene H Barbey C Rufer N. Enrichment of human CD4+ V(alpha)24/Vbeta11 invariant NKT cells in intrahepatic malignant tumors. J Immunol (2009) 182(8):5140–51. doi: 10.4049/jimmunol.0711086 19342695

[121] Tao L Wang S Kang G Jiang S Yine W Zong L. PD-1 blockade improves the anti-tumor potency of exhausted CD3. Oncoimmunology (2021) 10(1):2002068. doi: 10.1080/2162402X.2021.2002068 34777920 PMC8583083

[122] Cheng X Tan X Wang W Zhang Z Zhu R Wu M. Long-chain acylcarnitines induce senescence of invariant natural killer T cells in hepatocellular carcinoma. Cancer Res (2023) 83(4):582–94. doi: 10.1158/0008-5472.CAN-22-2273 36512635

[123] Jäkel CE Schmidt-Wolf IG. An update on new adoptive immunotherapy strategies for solid tumors with cytokine-induced killer cells. Expert Opin Biol Ther (2014) 14(7):905–16. doi: 10.1517/14712598.2014.900537 24673175

[124] Xiao YS Gao Q Xu XN Li YW Ju MJ Cai MY. Combination of intratumoral invariant natural killer T cells and interferon-gamma is associated with prognosis of hepatocellular carcinoma after curative resection. PloS One (2013) 8(8):e70345. doi: 10.1371/journal.pone.0070345 23940564 PMC3734128

[125] Li TT Sun J Wang Q Li WG He WP Yang RC. The effects of stereotactic body radiotherapy on peripheral natural killer and CD3. Hepatobiliary Pancreat Dis Int (2021) 20(3):240–50. doi: 10.1016/j.hbpd.2020.12.015 33454220

[126] Gao Y Guo J Bao X Xiong F Ma Y Tan B. Adoptive transfer of autologous invariant natural killer T cells as immunotherapy for advanced hepatocellular carcinoma: A phase I clinical trial. Oncologist (2021) 1126(11):e1919–30. doi: 10.1002/onco.13899 PMC8571770 34255901

[127] Mossanen JC Tacke F. Role of lymphocytes in liver cancer. Oncoimmunology (2013) 2(11):e26468. doi: 10.4161/onci.26468 24498546 PMC3906418

[128] Miyagi T Takehara T Tatsumi T Kanto T Suzuki T Jinushi M. CD1d-mediated stimulation of natural killer T cells selectively activates hepatic natural killer cells to eliminate experimentally disseminated hepatoma cells in murine liver. Int J Cancer (2003) 106(1):81–9. doi: 10.1002/ijc.11163 12794761

[129] Anson M Crain-Denoyelle AM Baud V Chereau F Gougelet A Terris B. Oncogenic β-catenin triggers an inflammatory response that determines the aggressiveness of hepatocellular carcinoma in mice. J Clin Invest (2012) 122(2):586–99. doi: 10.1172/JCI43937 PMC3266772 22251704

[130] Margalit M Shibolet O Klein A Elinav E Alper R Thalenfeld B. Suppression of hepatocellular carcinoma by transplantation of ex-vivo immune-modulated NKT lymphocytes. Int J Cancer (2005) 115(3):443–9. doi: 10.1002/ijc.20889 15688366

[131] Zhang C Cui X Feng L Han Z Peng D Fu W. The deficiency of FKBP-5 inhibited hepatocellular progression by increasing the infiltration of distinct immune cells and inhibiting obesity-associated gut microbial metabolite. J Gastrointest Oncol (2021) 12(2):711–21. doi: 10.21037/jgo-21-71 PMC8107620 34012660

[132] Liepelt A Wehr A Kohlhepp M Mossanen JC Kreggenwinkel K Denecke B. CXCR6 protects from inflammation and fibrosis in NEMO. Biochim Biophys Acta Mol Basis Dis (2019) 1865(2):391–402. doi: 10.1016/j.bbadis.2018.11.020 30476545

[133] Mossanen JC Kohlhepp M Wehr A Krenkel O Liepelt A Roeth AA. CXCR6 inhibits hepatocarcinogenesis by promoting natural killer T- and CD4. Gastroenterology (2019) 156(6):1877–1889.e4. doi: 10.1053/j.gastro.2019.01.247 30710528

[134] Eggert T Wolter K Ji J Ma C Yevsa T Klotz S. Distinct functions of senescence-associated immune responses in liver tumor surveillance and tumor progression. Cancer Cell (2016) 30(4):533–47. doi: 10.1016/j.ccell.2016.09.003 PMC7789819 27728804

[135] Braumüller H Wieder T Brenner E Aßmann S Hahn M Alkhaled M. T-helper-1-cell cytokines drive cancer into senescence. Nature (2013) 494(7437):361–5. doi: 10.1038/nature11824 23376950

[136] Chen J Gingold JA Su X. Immunomodulatory TGF-β signaling in hepatocellular carcinoma. Trends Mol Med (2019) 25(11):1010–23. doi: 10.1016/j.molmed.2019.06.007 31353124

